# Association of Medicare Beneficiary and Hospital Accountable Care Organization Alignment With Surgical Cost Savings

**DOI:** 10.1001/jamahealthforum.2022.4817

**Published:** 2022-12-22

**Authors:** Lindsey A. Herrel, Phyllis Yan, Parth Modi, Julia Adler-Milstein, Andrew M. Ryan, John M. Hollingsworth

**Affiliations:** 1 Dow Division of Health Services Research, Department of Urology, University of Michigan Medical School, Ann Arbor; 2 Department of Urology, University of Chicago, Chicago, Illinois; 3 Center for Clinical Informatics and Improvement Research, University of California, San Francisco, San Francisco; 4 Department of Urology, University of Michigan, Ann Arbor

## Abstract

**Importance:**

Although Medicare accountable care organizations (ACOs) account for half of program expenditures, whether ACOs are associated with surgical spending warrants further study.

**Objective:**

To assess whether greater beneficiary-hospital ACO alignment was associated with lower surgical episode costs.

**Design, Setting, and Participants:**

This retrospective cohort study was conducted between 2020 and 2022 using US Medicare data from a 20% random sample of beneficiaries. Individuals 18 years of age and older and without kidney failure who had a surgical admission between 2008 and 2015 were included. For each study year, distinction was made between beneficiaries assigned to an ACO and those who were not, as well as between admissions to ACO-participating and nonparticipating hospitals.

**Exposures:**

Time-varying binary indicators for beneficiary ACO assignment and hospital ACO participation and an interaction between them.

**Main Outcomes and Measures:**

Ninety-day, price-standardized total episode payments. Multivariable 2-way fixed-effects models were estimated.

**Results:**

During the study period, 2 797 337 surgical admissions (6% of which involved ACO-assigned beneficiaries) occurred at 3427 hospitals (17% ACO participating). Total Medicare payments for 90-day surgical episodes were lowest when ACO-assigned beneficiaries underwent surgery at a hospital participating in the same ACO as the beneficiary ($26 635 [95% CI, $26 426-$26 844]). The highest payments were for unassigned beneficiaries treated at participating hospitals ($27 373 [95% CI, $27 232-$27 514]) or nonparticipating hospitals ($27 303 [95% CI, $27 291-$27 314]). Assigned beneficiaries treated at hospitals participating in a different ACO and assigned beneficiaries treated at nonparticipating hospitals had similar payments (for participating hospitals, $27 003 [95% CI, $26 739-$27 267] and for nonparticipating hospitals, $26 928 [95% CI, $26 796-$27 059]). A notable factor in the observed differences in surgical episode costs was lower spending on postacute care services.

**Conclusions and Relevance:**

In this cohort study evaluating hospital and beneficiary ACO alignment and surgical spending, savings were noted for beneficiaries treated at hospitals in the same ACO. Allowing ACOs to encourage or require surgical procedures in their own hospitals could lower Medicare spending on surgery.

## Key Points

**Question:**

Is greater beneficiary-hospital Medicare accountable care organization (ACO) alignment associated with lower surgical episode costs?

**Findings:**

In this cohort study of 2 797 337 surgical admissions at 3427 hospitals, total payments for 90-day surgical episodes were lowest when ACO-assigned beneficiaries underwent surgery at a hospital participating in the same ACO as theirs, whereas payments were highest for unassigned beneficiaries treated at ACO-participating hospitals or nonparticipating hospitals.

**Meaning:**

Findings of this study suggest that allowing ACOs to encourage or require surgical procedures in their own hospitals could be associated with lower Medicare spending on surgery, which currently accounts for half of all program expenditures.

## Teaser

This cohort study analyzes US Medicare data to examine surgical episode costs before and after accountable care organization implementation among assigned and unassigned beneficiaries treated at participating and nonparticipating hospitals.

## Introduction

Medicare payments for US surgical care are substantial, exceeding $120 billion annually and accounting for approximately half of program expenditures.^1^ Given the outsized contribution of surgery to overall spending, it serves as a logical target for savings among hospitals participating in Medicare’s largest accountable care organization (ACO) model—the Shared Savings Program (SSP). However, a 2019 evaluation^2^ of the SSP suggested that surgical episode costs for major inpatient procedures are comparable at ACO-participating and nonparticipating hospitals.

To inform SSP design improvements, a better understanding is needed of what may be hampering realization of savings around surgery at ACO-participating hospitals. A possible explanation, but one that has not been examined empirically, relates to the open nature of SSP ACO contracting networks. Particularly, Medicare fee-for-service beneficiaries are free to seek care from whomever they want regardless of their ACO assignment and a facility’s participation status.^3^

Consider an older adult with osteoarthritis of the hip who seeks joint replacement surgery. If she is assigned to an SSP ACO but chooses to have her arthroplasty procedure performed at a hospital that does not participate in the program, her ACO’s care coordination capabilities—one of the primary means for controlling costs after discharge—could be limited, as the ACO cannot directly affect what occurs at the hospital.^4^ If instead she is unassigned to an SSP ACO and chooses to have surgery at an SSP ACO-participating hospital, that hospital might withhold from her extra resources, such as discharge planners and clinical navigators that come at an expense for the hospital, because the hospital’s ACO would not benefit if reductions in her surgical episode costs are achieved.

These 2 scenarios illustrate the interdependent dynamics that may be operating at the individual and organizational levels to produce different magnitudes of savings. Following an SSP launch, we would expect the greatest savings for assigned beneficiaries receiving their surgical care at participating hospitals, whereas unassigned beneficiaries treated at nonparticipating hospitals would realize little, if any, savings. The other 2 scenarios—in which 1 of the 2 dimensions is aligned—would likely fall in the middle. To test these hypotheses and generate findings that can help guide ongoing ACO design decisions, we analyzed national Medicare data by examining surgical episode costs before and after ACO implementation among assigned and unassigned beneficiaries treated at participating and nonparticipating hospitals.

## Methods

### Data Sources and Study Population

For this cohort study (conducted between 2020 and 2022), we used Medicare claims (2008 to 2015) from a 20% random US sample of Medicare beneficiaries, including data from the Carrier, Denominator, Outpatient, and Medicare Provider and Analysis Review Research Identifiable Files. Our study population included beneficiaries 18 years of age and older and without kidney failure who had an acute care hospital admission with a surgical diagnosis-related group (DRG) over the study interval. DRGs are classified by Medicare as surgical or medical depending on the primary diagnosis and whether a surgical procedure is performed. We used this preexisting classification to identify acute care hospital admissions with a surgical DRG as defined by Medicare and the Inpatient Prospective Payment System. We used racial and ethnic categories provided within the Medicare data sets. In these data, race is categorized as Black, White, and other (ie, American Indian or Alaska Native, Asian or Pacific Islander, Hispanic, unknown, and other) using information obtained by the Social Security Administration and provided to the Centers for Medicare & Medicaid Services for enrollment data. An additional algorithm is applied to these data that further identifies beneficiaries as Hispanic or Asian and provides categorization of ethnicity. We required continuous Medicare Parts A and B enrollment for 1 year prior to the index admission and 90 days after discharge to ensure ascertainment of all relevant medical claims. We further required that beneficiaries received at least 1 primary care service (Healthcare Common Procedure Coding System codes 99201 through 99215, 99304 through 99350, G0402, G0438, and G0439) during the year of admission furnished by an ACO professional to ensure that all beneficiaries in this sample were eligible for ACO assignment.^5,6^ We excluded patients who were missing an admission or discharge date and those treated at hospitals in Maryland or Puerto Rico, since they are not a part of Medicare’s Inpatient Prospective Payment System. This study follows the Strengthening the Reporting of Observational Studies in Epidemiology (STROBE) reporting guideline for cohort studies.

### Determining Beneficiary Assignment and Hospital Participation

We determined whether a beneficiary in a given study year was assigned to an ACO using the SSP Beneficiary-level Research Identifiable File (2012 to 2015). With data from Torch Insight,^7^ we determined whether the hospital at which a beneficiary underwent surgery participated in an SSP ACO. Updated regularly through public records and interviews, this validated database has over 30 fields of information on ACOs. Alignment of beneficiary and hospital ACO participation occurs when an assigned beneficiary has surgery at a hospital participating in the same ACO to which the beneficiary is assigned. Table 1 presents concepts of beneficiary ACO assignment, hospital ACO participation, and beneficiary-hospital ACO alignment. Consistent with prior work,^8^ we defined hospital participation as ownership or nonownership affiliation between the hospital and an SSP ACO.

**Table 1.** Beneficiary Assignment, Hospital Participation, and Alignment Definitions

Hospital ACO	Beneficiary ACO assignment		Beneficiary-hospital ACO alignment
	Unassigned	Assigned	
Participating	Unassigned beneficiary receives surgery in ACO-participating hospital	ACO-assigned beneficiary receives surgery in hospital participating in same ACO as beneficiary	Yes
	Unassigned beneficiary receives surgery in ACO-participating hospital	ACO-assigned beneficiary receives surgery in hospital participating in a different ACO from beneficiary	No
Nonparticipating	Unassigned beneficiary receives surgery in nonparticipating hospital	ACO-assigned beneficiary receives surgery in nonparticipating hospital	Not applicable

Abbreviation: ACO, accountable care organization.

### Measuring Surgical Episode Costs

Our primary outcome of interest was the total payment for a surgical episode. We defined surgical episodes as starting from the beneficiary’s admission date for surgery and extending to 90 days postdischarge. This length was informed by Medicare’s current value-based payment models.^9,10^ We summed all Medicare payments made on a beneficiary’s behalf that fell within this episode window. We further classified payments into those for the index admission, professional services, readmissions, and postacute care. We used established methods to price costs of standardized payments, removing geographic and other adjustments unrelated to the services delivered.^11^ Further, we adjusted payments to 2015 US dollars. Through the process of price standardization, differences in spending were largely reflective of differences in use.

### Statistical Analysis

We performed all statistical analyses using SAS, version 9.4 (SAS Institute Inc) and Stata, version 15.1 (StataCorp LLC). We used 2-tailed tests with a significance level of *P* = .05. This study was deemed exempt from oversight and patient consent by the University of Michigan institutional review board as a secondary analysis of deidentified data.

We began by characterizing ACO-assigned and unassigned beneficiaries over a variety of patient factors (age, sex, race and ethnicity, dual eligibility, socioeconomic status [SES; assessed with a zip-code based composite measure], and level of comorbid illness [determined with the Hierarchical Condition Category methodology]).^12,13^ Next, we characterized ACO-participating and nonparticipating hospitals. Based on data from the American Hospital Association Annual Survey,^14^ we examined differences with respect to their number of beds, teaching status, urbanicity, for-profit status, and geographic location. Through the Centers for Medicare & Medicaid Services’ Impact File, we also evaluated hospitals with respect to their Medicaid share.

To assess beneficiary-hospital alignment and total payments, we estimated a 2-way fixed-effects model using time-varying patient assignment to and hospital participation in an SSP ACO. With the index admission serving as our unit of analysis, we fit a series of multivariable linear regression models for our primary (total payments) and secondary outcomes (each of the component payments described above). Admissions with missing data were excluded from analyses. We included in our models time-varying binary indicators for beneficiary ACO assignment and hospital ACO participation, as well as an interaction between the two. These indicators had a value of ‘1’ if a beneficiary was assigned to or a hospital participated in an ACO during a given study year. They had a value of ‘0’ otherwise.

We controlled for the various patient factors described above and the DRG associated with the beneficiary’s index admission by including these as fixed effects in our models. Age was categorized into 6 categories (aged <65, 65-69, 70-74, 75-79, 80-84, and ≥85 years) to account for the nonlinear association between age and total payments. In addition, we included quarter, year, and hospital fixed effects. We used a cluster robust variance estimator to account for the association of admissions within hospitals.^15^ To express the association between surgical episode costs and beneficiary-hospital alignment, we estimated adjusted total and component payments for beneficiaries assigned and not assigned to ACOs and hospitals participating and not participating in ACOs as estimated margins.^16^

To see if results differed when an assigned beneficiary underwent surgery at a hospital that was part of their assigned ACO vs at a hospital that was part of a different ACO, we examined whether total payments differed between these 2 groups. In particular, we repeated our models, including indicators for beneficiaries whose index admission occurred at a hospital participating in the same ACO to which they were assigned and for those whose index admission occurred at a hospital participating in a different ACO. We then estimated the difference between the effects of these 2 groups.

Finally, to test the robustness of our findings, we conducted several sensitivity analyses. Given prior literature^6^ suggesting an association between ACO performance and organizational structure, we repeated our models with indicators that distinguished between physician-led and hospital-led organizations and hospital-physician partnerships. To allow for learning outcomes, we re-estimated the association between surgical costs and ACO implementation, accounting for years of SSP experience. Because of possible selection effects between early and late model adopters, we reran the primary analysis by adjusting for ACO contract start date.^17,18^ To follow up on our findings of a lower proportion of patients with dual eligibility and lower SES being assigned to an ACO and having these differences potentially affect our findings, we estimated overall and postacute care spending for these subgroups in particular.

## Results

During the study period, there were 2 797 337 surgical index admissions (6% of which involved ACO-assigned beneficiaries) at 3427 hospitals (17% ACO participating) involving 2 054 274 unique beneficiaries. Table 2 compares patient characteristics between assigned and unassigned beneficiaries on admission. While the 2 groups were clinically comparable with respect to age, sex, race and ethnicity, and level of comorbid illness, admissions for unassigned beneficiaries occurred more frequently among those simultaneously enrolled in Medicaid and Medicare (ie, dual eligibility) and those from the lowest socioeconomic stratum. Table 3 compares ACO-participating and nonparticipating hospitals. Participating hospitals were more often larger, had a teaching mission, and were located in an urban area. Nonparticipating hospitals were more often for profit, located in the Southern region, and had a higher proportion of disproportionate share payments.

**Table 2.** Differences Between ACO-Assigned and Unassigned Beneficiaries

Characteristic	Medicare beneficiaries, %	
	Assigned	Unassigned
No. of admissions	172 131	2 625 206
Age, median (IQR), y	74 (68-80)	74 (68-81)
Sex		
Male	44	44
Female	56	56
Race		
Black	7	7
White	86	86
Other^a^	7	7
Ethnicity		
Hispanic	4	4
Non-Hispanic	96	96
Dual eligibility	15	19
No. of HCCs, median (IQR)	2 (1-3)	2 (1-3)
Socioeconomic status^b^		
High	42	33
Medium	33	33
Low	25	34

Abbreviations: ACO, accountable care organization; HCC, Hierarchical Condition Category.

^a^ Other includes American Indian or Alaska Native, Asian or Pacific Islander, Hispanic, unknown, and other. Racial and ethnic categories are provided within the Medicare data sets.

^b^ Assessed with a zip-code based composite measure.

**Table 3.** Differences Between ACO-Participating and Nonparticipating Hospitals

Characteristic	Hospitals, %	
	Participating	Nonparticipating
No. of hospitals	586	2841
No. of beds		
<200	49	66
200 to 349	24	19
350 to 499	12	8
≥500	15	7
Teaching hospital	13	6
Urban	97	90
For-profit status	8	28
US Census Bureau region		
South	30	45
Northeast	27	13
West	10	21
Midwest	33	21
Highest tercile of disproportionate share payments	31	40

Abbreviation: ACO, accountable care organization.

**Figure 1.**
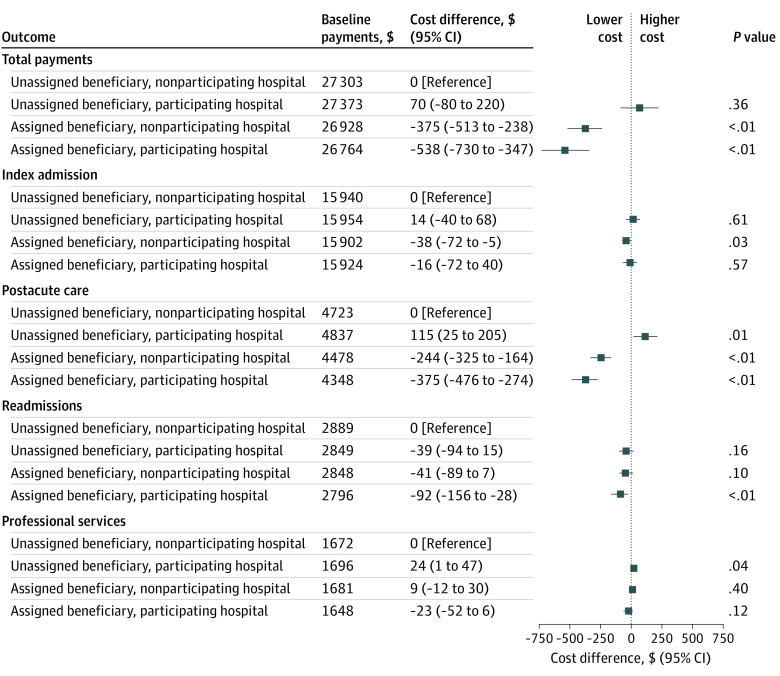
Beneficiary-Hospital Alignment and Total and Component Payments In results of multivariable analysis, adjusted total payments were lowest for the assigned beneficiaries who received treatment from a participating hospital, followed by those for assigned beneficiaries treated at a nonparticipating hospital. A notable factor in the observed differences in surgical episode costs was lower spending on postacute care services. Nonparticipating hospital indicates that a hospital was not part of the Medicare accountable care organization, whereas a participating hospital indicates that the hospital was part of the Medicare accountable care organization.

Figure 1 shows results from the multivariable analysis. Adjusted total Medicare payments for 90-day surgical episodes were lowest for ACO-assigned beneficiaries who received treatment from an ACO-participating hospital ($26 764 [95% CI, $26 580-$26 948], on average, per surgical episode), followed by those for assigned beneficiaries treated at a nonparticipating hospital ($26 928 [95% CI, $26 796-$27 059]). Both totals were significantly lower than the average for unassigned beneficiaries who underwent surgery at nonparticipating hospitals ($27 303 [95% CI, $27 291-$27 314]; *P* < .001 for each comparison). In fact, total payments for this last group were comparable to those for unassigned beneficiaries treated at participating hospitals ($27 373 [95% CI, $27 232-$27 514]; *P* = .36).

Figure 1 also highlights that the most notable factor of the observed differences in surgical episode costs was lower spending on postacute care services. Admissions among assigned beneficiaries to participating and nonparticipating hospitals were associated with adjusted payments for postacute care services that were, on average, $375 [95% CI, $275-$476] and $245 [95% CI, $164-$325] lower, respectively, than those for unassigned beneficiaries treated at nonparticipating hospitals (*P* < .001 for each difference). Small but significant differences in spending on the index admission were also noted, with assigned beneficiaries treated at nonparticipating hospitals having the lowest adjusted payments ($15 902 [95% CI, $15 869-$15 934] vs $15 940 [95% CI, $15 936-$15 944] for unassigned beneficiaries treated at nonparticipating hospitals; *P* = .03). Details about unadjusted spending by exposure group and study year are available in eTable 1 and eTable 2 in Supplement 1.

**Figure 2.**
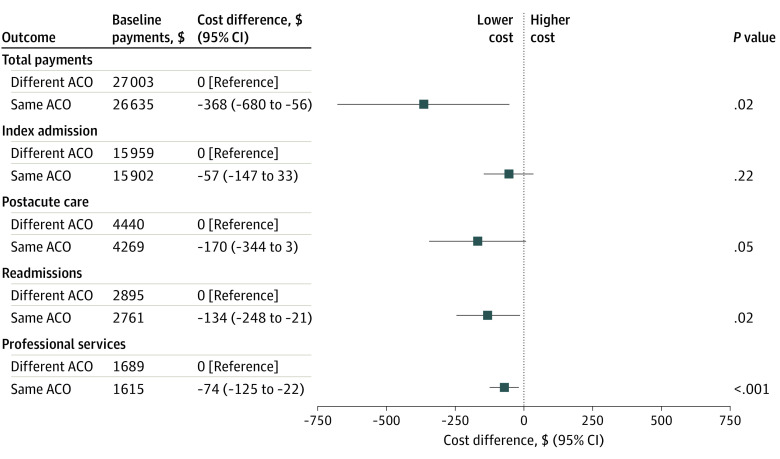
Differences in Total and Component Payments Among ACO-Assigned Beneficiaries Treated at a Hospital Participating in Their Assigned ACO vs a Different ACO Results of multivariable analysis. Savings around the surgical episode among ACO-assigned beneficiaries treated at an ACO-participating hospital were greater when their admission occurred at a facility in the same ACO to which they were assigned vs a different one. Lower spending on professional services and readmissions was a factor in the observed difference. ACO indicates accountable care organization.

Figure 2 shows that savings around the surgical episode among ACO-assigned beneficiaries treated at an ACO-participating hospital were greater when their admission occurred at a facility in the same ACO to which they were assigned vs a different one ($26 635 [95% CI, $26 426-$26 844] vs $27 003 [95% CI, $26 739-$27 267]; *P* = .02). Indeed, surgical episode costs for assigned beneficiaries treated at a hospital participating in a different ACO than their own most closely approximated those of assigned beneficiaries treated at a nonparticipating hospital ($26 928 [95% CI, $26 796-$27 059]). Lower spending on professional services (−$74 [95% CI, −$125 to −$22]) and readmissions (−$134 [95% CI, -$248 to -$21]) may explain the observed difference.

Finally, findings from our main analysis persisted in sensitivity analyses that accounted for ACO differences in organizational structure, years of program experience, and contract start date. Our subgroup analyses showed no significant differences in overall or postacute care spending according to SES strata. However, we found higher overall spending for dual-eligible ($28 872 [95% CI, $28 803-$28 941]) compared with nondual-eligible beneficiaries ($26 922 [95% CI, $26 906-$26 937]). We further identified higher spending within postacute care for dual-eligible beneficiaries ($5825 [95% CI, $5782-$5868] vs $4459 [95% CI, $4450-$4469] for nondual-eligible beneficiaries.

## Discussion

In this cohort study examining hospital and beneficiary ACO alignment and surgical spending, we have 3 principal findings. First, surgical episode costs were lowest when ACO-assigned beneficiaries underwent surgery at ACO-participating hospitals (and even more so if it was the specific ACO to which the beneficiary was assigned), followed by those for assigned beneficiaries treated at nonparticipating hospitals. Second, these savings were associated with lower spending on postacute care services that did not come at the expense of increased readmission payments. Third, not only were surgical episode costs highest for unassigned beneficiaries, but they were also comparable whether surgery occurred at a participating or nonparticipating hospital.

Results from previous studies examining the ability of the ACOs to reduce surgical episode costs are conflicting. Nathan and colleagues^2^ examined a cohort of over 340 000 patients enrolled in Medicare who underwent 6 common elective procedures at 427 ACO-participating hospitals. They found no difference in total payments for surgical episodes among these patients compared with matched controls treated at nonparticipating hospitals. More recently, Modi and colleagues^19^ analyzed claims data from a national sample of Medicare beneficiaries, evaluating for differences in spending on surgery between those assigned and unassigned to an SSP ACO. For the subset whose procedure was performed on an inpatient basis, total payments for surgical episodes were nearly $1000 lower, on average, for assigned beneficiaries. Our analysis helps reconcile the seemingly contradictory findings from these 2 studies by assessing the interaction between the hospital ACO participation status and the beneficiary ACO assignment.

Specifically, our analysis suggests that the individual beneficiary’s assignment status matters, and participating hospitals may be systematically discriminating between surgical patients based on this factor. One possible reason for this behavior is that the resources needed by hospitals to manage a patient’s safe transition after surgery from the acute care setting to the community are scarce and costly.^20^ Thus, participating hospitals might make business decisions to concentrate their care coordination efforts on surgical patients for whom they are incentivized financially to reduce discretionary spending. Further support for this possibility comes from our subgroup analysis of ACO-assigned beneficiaries who received treatment at an ACO-participating hospital. Namely, we found that surgical episode costs among beneficiaries admitted to a facility participating in an ACO different from the one to which they were assigned were higher than those for an ACO’s beneficiaries treated at one of its participating hospitals. The former’s total payments were more comparable to those of assigned beneficiaries treated at a nonparticipating hospital. The challenge with a participating hospital taking such an approach is that identifying for which beneficiaries it is responsible may be difficult. Prior to 2019, assignment occurred retrospectively (after Medicare claims were processed at the performance year’s end).

Our analysis also suggests the ability of SSP ACOs to lower surgical episode costs even when procedures are performed at nonparticipating hospitals, albeit an attenuated effect. This finding is consistent with empirical work^21^ evaluating skilled nursing facility use among assigned beneficiaries after discharge, finding similar decreases for organizations regardless of whether there was a formal hospital association. There are at least 2 mechanisms through which an ACO could be a factor in the inpatient surgical care (and, therefore, decisions about postdischarge services) that its assigned beneficiaries receive at a nonparticipating hospital. For one, surgeons who staff the surgical admissions at a given hospital may be part of the ACO’s contracting network. Alternatively, an ACO could partner informally with surgical practices whose surgeons are credentialed at the hospital.

Our analysis has important policy implications. In an effort to rein in health care spending growth, the Centers for Medicare & Medicaid Services is trying to shift delivery systems toward value-based care. The SSP represents arguably the most substantial effort to make this shift a reality. As a major cost center for Medicare, surgical care is a logical target for ongoing and planned ACO initiatives and should be a priority area for ACOs to evaluate and optimize spending. Our analysis indicates that beyond reductions in postacute care use for surgical patients, when there is beneficiary-hospital alignment, ACOs can achieve greater savings through less intensive inpatient care and improved care coordination (ie, reduced spending on professional services and readmissions). As such, policy makers may consider ways of reducing out-of-network surgical care, including development of appropriate incentives for beneficiaries and ACOs alike. Expanding programs such as the ACO Beneficiary Incentive Program, which allows certain ACOs to pay beneficiaries to receive in-network primary care, could help facilitate this objective. Obviously, this endeavor would need to be balanced with preservation of patient choice—a defining characteristic of the SSP. Maintaining patient choice and timely access to high-quality surgical care is particularly important for patients with rare and comorbid conditions, as well as those who require highly specialized surgical care that may not be available at all centers. Balancing and monitoring access, quality, and spending will be crucial as new value-based programs are developed and implemented. Our findings demonstrating increased spending for dual-eligible beneficiaries both overall and in postacute care suggest that focused support for dual-eligible beneficiaries (eg, care navigation) may be an opportunity for intervention. Another option would be to combine ACO participation with other hospital-wide strategies to limit wasteful spending around surgical episodes (eg, bundled payment programs) since a hospital’s ACO contract may account for only a small proportion of admissions to it. Finally, optimizing the patient experience within an ACO provides an opportunity to harmonize patient choice and delivery of high quality in-network (ie, aligned) care.

### Limitations

Our findings should be interpreted within the context of several limitations. First and foremost, the SSP is a completely voluntary program, and the participation decision is nonrandom. While we made every effort to account for differences between participants and nonparticipants with a broad set of beneficiary and organizational controls and hospital fixed effects, we cannot exclude the possibility that the savings we observed were associated with the decisions of high-cost facilities (and their patient panels) to exit the SSP.^22^ Similarly, our models are adjusted for measurable confounders; however, the possibility of residual unmeasured confounding of our results remains a limitation. Second, we examined claims data through 2015, and the generalizability of our findings to more contemporary years is unclear. This factor is important because the SSP underwent a major overhaul in 2019. As part of the so-called “Pathways to Success,” participants were required to take on downside financial risk more quickly, which may have altered their approach to surgical care.^23^ Our findings may therefore underestimate the consequences of beneficiary-hospital ACO alignment if a substantial shift in focus onto surgical care has occurred. Given this possibility, any alterations have likely been modest given only slight increases in net per-beneficiary savings since the launch of Pathways to Success.^24^ Despite a lack of data from the last few years, we believe our findings remain important for ACO stakeholders (eg, hospitals and beneficiaries) as well as for policy makers who are continually striving to improve engagement and outcomes associated with the ACO programs. Last, we did not examine surgical quality outcomes, such that further studies are needed to determine if our findings extend to this essential component of accountable care.

## Conclusions

In this cohort study examining hospital and beneficiary ACO alignment and surgical spending, we found that SSP ACOs were able to reduce surgical spending more when there was greater beneficiary and hospital alignment (around $700 per episode). Taken together, allowing ACOs to encourage or require their assigned beneficiaries to undergo surgical procedures in their own hospital(s) could lower Medicare spending on surgery.

## Notes

Supplement 1. **eTable 1.** Unadjusted Spending by Exposure Group **eTable 2.** Unadjusted Spending by Study Year

Supplement 2.
Data Sharing Statement
