# Supplementary material for: Association of Medicare Beneficiary and Hospital Accountable Care Organization Alignment With Surgical Cost Savings
Source: JAMA Health Forum. 2022 Dec 22;3(12):e224817. doi: 10.1001/jamahealthforum.2022.4817 (PMC9857079; doi:10.1001/jamahealthforum.2022.4817)
Supplement: Supplement 1. — eTable 1. Unadjusted Spending by Exposure Group eTable 2. Unadjusted Spending by Study Year [file jamahealthforum-e224817-s001.pdf]

Supplemental Online Content

Herrel LA, Yan P, Modi P, Adler-Milstein J, Ryan AM, Hollingsworth JM. Association of Medicare beneficiary and hospital accountable care organization alignment with surgical cost savings. *JAMA Health Forum.* 2022;3(12):e224817. doi:10.1001/jamahealthforum.2022.4817

eTable 1. Unadjusted Spending by Exposure Group

eTable 2. Unadjusted Spending by Study Year

This supplemental material has been provided by the authors to give readers additional information about their work.

© 2022 Herrel LA et al. *JAMA Health Forum*.

eTable 1. Unadjusted Spending by Exposure Group

		Beneficiary ACO Assignment		
		**Unassigned**	**Assigned**	**Beneficiary-Hospital ACO Alignment**
**Hospital ACO**	**Participating**	Unassigned beneficiary receives surgery in ACO participating hospital **(Total average spending: 27,965)**	ACO assigned beneficiary receives surgery in hospital participating in **same** ACO as beneficiary **(Total average spending: 25,851)**	Yes
		Unassigned beneficiary receives surgery in ACO participating hospital **(Total average spending: 27,965)**	ACO assigned beneficiary receives surgery in hospital participating in a **different** ACO from beneficiary **(Total average spending: 26,801)**	No
	**Non-participating**	Unassigned beneficiary receives surgery in non-participating hospital **(Total average spending: 27,309)**	ACO assigned beneficiary in non-participating hospital **(Total average spending: 26,445)**	Not Applicable

© 2022 Herrel LA et al. *JAMA Health Forum*.

eTable 2. Unadjusted Spending by Study Year

**Study year**	**Number of observations**	**Total average spending**
2008	404,020	27,168
2009	387,216	27,835
2010	377,311	27,737
2011	366,630	27,935
2012	341,996	26,914
2013	335,619	26,680
2014	326,813	26,960
2015	257,732	26,677

© 2022 Herrel LA et al. *JAMA Health Forum*.
