# Supplementary material for: Association of Medicare Beneficiary and Hospital Accountable Care Organization Alignment With Surgical Cost Savings
Source: JAMA Health Forum. 2022 Dec 22;3(12):e224817. doi: 10.1001/jamahealthforum.2022.4817 (PMC9857079; doi:10.1001/jamahealthforum.2022.4817)
Supplement: Supplement 2. — Data Sharing Statement [file jamahealthforum-e224817-s002.pdf]

Data Sharing Statement

Data
Data available: No

Additional Information
Explanation for why data not available: No additional data is available outside of the published and supplemental data files due to patient confidentiality restrictions with Medicare data.
